# DISPARITIES IN ELECTION, ACCESS, AND OUTCOMES IN MEDICARE END-OF-LIFE CARE: A NATIONAL STUDY

**DOI:** 10.1093/geroni/igad104.1549

**Published:** 2023-12-21

**Authors:** Thomas Christian, Michael Plotzke, Mariana Sarango Cancel, Catherine Hersey, Zinnia Harrison

**Affiliations:** Abt Associates, Cambridge, Massachusetts, United States; Abt Associates, St. Louis, Missouri, United States; Abt Associates, Cambridge, Massachusetts, United States; Abt Associates, Cambridge, Massachusetts, United States; Abt Associates, Inc., Rockville, Maryland, United States

## Abstract

We examined whether end-of-life care racial disparities persist even within groups with similar geographic access and health care options. We reviewed calendar year (CY)2021 fee-for-service Medicare claims to determine if a beneficiary ever: elected hospice, had an end-of-life care conversation with their physician, and/or received advanced care planning services. We obtained beneficiary characteristics from Medicare administrative records and used Consumer Assessment of Health Providers and Systems Hospice star ratings to identify poorer quality hospices (one- or two-stars out of five). As a measure of the quality-of-care processes beneficiaries received, we calculated Hospice Visits in the Last Days of Life (HVLDL). Using logistic regression, we calculated Adjusted Odds Ratios (AORs) and 95% Confidence Intervals (CIs) to characterize the association between race/ethnicity and end-of-life services utilization. Relative to white decedents, beneficiaries were less likely to elect hospice if they were black [AOR 0.67 95% CI 0.65-0.69], Asian [AOR 0.62 95% CI 0.59-0.66] or Hispanic [AOR 0.76 95% CI 0.72-0.81]. Additionally, non-white beneficiaries more often received care from poorer quality hospices than the white beneficiaries residing in their same zip code (black AOR 1.26 95% CI 1.21-1.31, Asian AOR 1.21 95% CI 1.14-1.29, Hispanic AOR 1.16 95% CI 1.09-1.24). In such poorer quality hospices, non-white beneficiaries have lower HVLDL rates (white 48.5%, black 40.3%, Asian 42.6%, Hispanic 45.8%). We found no substantial racial/ethnic disparities in recorded advance care plans or end-of-life conversations. CMS should continue to monitor trends in hospice utilization and ensure all beneficiaries receive equal and adequate care.

